# How Does Antimicrobial Stewardship Affect Inappropriate Antibiotic Therapy in Urological Patients?

**DOI:** 10.3390/antibiotics9020063

**Published:** 2020-02-06

**Authors:** Atsushi Uda, Katsumi Shigemura, Koichi Kitagawa, Kayo Osawa, Kenichiro Onuma, Shigeaki Inoue, Joji Kotani, Yonmin Yan, Yuzo Nakano, Tatsuya Nishioka, Ikuko Yano, Takayuki Miyara, Masato Fujisawa

**Affiliations:** 1 Department of Infection Control and Prevention, Kobe University Hospital, Kobe 650-0017, Japan; a-uda@umin.ac.jp (A.U.); onumak@med.kobe-u.ac.jp (K.O.); miyarat@med.kobe-u.ac.jp (T.M.); 2 Department of Pharmacy, Kobe University Hospital, Kobe 650-0017, Japan; tnishi@med.kobe-u.ac.jp (T.N.); iyano@med.kobe-u.ac.jp (I.Y.); 3 Division of Infectious Diseases, Department of Public Health, Kobe University Graduate School of Health Sciences, Kobe 654-0142, Japan; ko1.kitgwa@gmail.com (K.K.); osawak@kobe-u.ac.jp (K.O.); 4 Division of Urology, Kobe University Graduate School of Medicine, Kobe 650-0017, Japan; yym1112@gmail.com (Y.Y.); yznakano@med.kobe-u.ac.jp (Y.N.); masato@med.kobe-u.ac.jp (M.F.); 5 Division of Advanced Medical Science, Kobe University Graduate School of Science, Technology and Innovation, Kobe 657-8501, Japan; 6 Department of Medical Technology, Kobe Tokiwa University, Kobe 653-0838, Japan; 7 Department of Disaster and Emergency Medicine, Kobe University Graduate School of Medicine, Kobe 650-0017, Japan; inoues@med.kobe-u.ac.jp (S.I.); kotanijo0412@gmail.com (J.K.)

**Keywords:** antimicrobial stewardship team, intervention, urological patient

## Abstract

Antimicrobial stewardship teams (ASTs) have been well-accepted in recent years; however, their clinical outcomes have not been fully investigated in urological patients. The purpose of this study was to evaluate the outcomes of intervention via a retrospective review of urological patients, as discussed in the AST meetings, who were treated with broad-spectrum antibiotics between 2014 and 2018 at the Department of Urology, Kobe University Hospital in Japan. Interventions were discussed in AST meetings for patients identified by pharmacists as having received inappropriate antibiotic therapy. The annual changes in numbers of inappropriate medications and culture submissions over five years at the urology department were statistically analyzed. Among 1,033 patients audited by pharmacists, inappropriate antibiotic therapy was found in 118 cases (11.4%). The numbers of inappropriate antibiotic use cases and of interventions for indefinite infections had significantly decreased during the study period (*p* = 0.012 and *p* = 0.033, respectively). However, the number of blood and drainage culture submissions had significantly increased (*p* = 0.009 and *p* = 0.035, respectively). Our findings suggest that urologists have probably become more familiar with infectious disease management through AST intervention, leading to a decrease in inappropriate antibiotic use and an increase in culture submissions.

## 1. Introduction

The main goal of antimicrobial stewardship (AS) is to decrease the incidence of antimicrobial resistance (AMR) [1]. Healthcare professionals and even national governments have called for action to prevent the increase of AMR by the appropriate use of broad-spectrum antibiotics, such as carbapenems, antibiotics with anti-*Pseudomonas* activity and anti-methicillin-resistant *Staphylococcus aureus* (MRSA) agents, based on reliable publications or guidelines. The concept of AS has spread globally, and the Japanese government has recently offered an additional medical fee for hospitals having the established AS team (AST) system [2]. Basically, the AST consists of physicians, pharmacists, clinical microbiologists, and nurses specialized in infectious disease and antimicrobial agents [3]. They are often certified infection control specialists such as infection control doctors (ICDs). Most ICDs are internal medical doctors (physicians) who are familiar with medications, but not with surgical interventions such as drainages [4]. Therefore, surgeons need to be included in the ASTs as well as in infection control teams (ICTs), to achieve the best outcomes for surgical patients [5]. Especially in the case of urinary tract infection (UTI), physicians often make late decisions to carry out drainage or use of invalid antibiotics that possibly generate resistance in bacteria. Furthermore, as urologists sometimes use invalid broad-spectrum antibiotics, collaboration among several professionals in ASTs is urgently needed. This study investigated patients, who were admitted and treated with broad-spectrum antibiotics in the urological ward, and compared the changes in the numbers of inappropriately prescribed medications and culture submissions over five years.

## 2. Materials and Methods 

### 2.1. Patients

The present study included infectious disease patients admitted in the urological ward in Kobe University Hospital, who were discussed in the AST meetings held between 2014 and 2018. In our AST meetings, these patients were reviewed for the appropriateness of broad-spectrum antibiotic use, including antibiotic medication or whether cultures had to be obtained for antibiotic selection. This study was approved by the Kobe University Graduate School of Health Sciences Institutional Review Board (IRB) (No. 472-5). 

### 2.2. Antimicrobial Stewardship Team (AST) Meetings

In March 2010, Kobe University Hospital started AST meetings which was called as the “Big Gun Project” in our hospital and focused on weekly prospective audit and feedback [6]. The main objectives of this project team were to promote the appropriate use of antimicrobial agents and to prevent the emergence of AMR. We defined antipseudomonal antibiotics and anti-MRSA agents as targeted, intravenous, broad-spectrum antibiotics in this meeting. Pharmacists in the AST audited all patients who used targeted antimicrobial agents in the previous week, and inappropriate (unnecessary) use or unsubmited culture cases were extracted for intervention. When the cases were discussed in the AST meeting, pharmacists recommended pharmaceutical interventions, such as another antimicrobial agent, optimization of the duration of administration or culture submissions, to the doctors. In these AST meetings, pharmacists, clinical microbiologists, nurses, and infectious disease physicians, including a urologist, collaborated to optimize the use of broad-spectrum antimicrobial agents for each patient. We found that this project was highly effective in reducing the use of antipseudomonal antibiotics [6].

### 2.3. Infectious Diseases and Reasons for Intervention

The medical records of patients, who were discussed in the AST meeting, were examined for urological infectious diseases and reasons for intervention. The types of disease that required drainage were as follows: pyelonephritis, peritonitis, refractory urachal abscess, renal abscess, appendicitis with abscess, perineum and penile abscess, pelvic abscess, Fournier’s gangrene, pneumonia or liver abscess, renal cyst, and retroperitoneal abscess. Diseases other than the aforementioned ones had only one case during the entire study period.

### 2.4. Antibiotics Prescribed

The AST investigated the following intravenous antibiotics in the meetings: (1) Antipseudomonal penicillins—piperacillin and piperacillin/tazobactam; (2) Monobactam—aztreonam; (3) Antipseudomonal cephalosporins—ceftazidime, cefepime, and cefozopran; (4) Carbapenems—meropenem, and doripenem; (5) Fluoroquinolones—ciprofloxacin, levofloxacin, and pazufloxacin; (6) Aminoglycosides—amikacin, tobramycin, and gentamicin; (7) Anti-MRSA agents—vancomycin, teicoplanin, linezolid, and daptomycin; (8) Other—colistin.

### 2.5. Microbiological Culture Collections

The data for blood, urine, or drainage culture collections of the patients admitted in the urological ward were obtained from microbiology laboratory records (blood culture sets/1000 patient-days, urine or drainage cultures/1000 patient-days).

### 2.6. Statistical Analysis

Appropriateness of cases, disease, selected antimicrobials, reasons for intervention, and microbiological cultures for each year were compared across a five-year period. Single regression analysis was performed to determine the association between the years and each data category by using EZR (Saitama Medical Center, Jichi Medical University, Saitama, Japan). In case of any change in the number of patients between the years, Spearman’s correlation coefficient by rank was used. Statistical analysis was performed if more than 9 cases were included. The threshold for statistical significance was set at *p* < 0.05. 

## 3. Results

### 3.1. Patients

**Figure 1:**
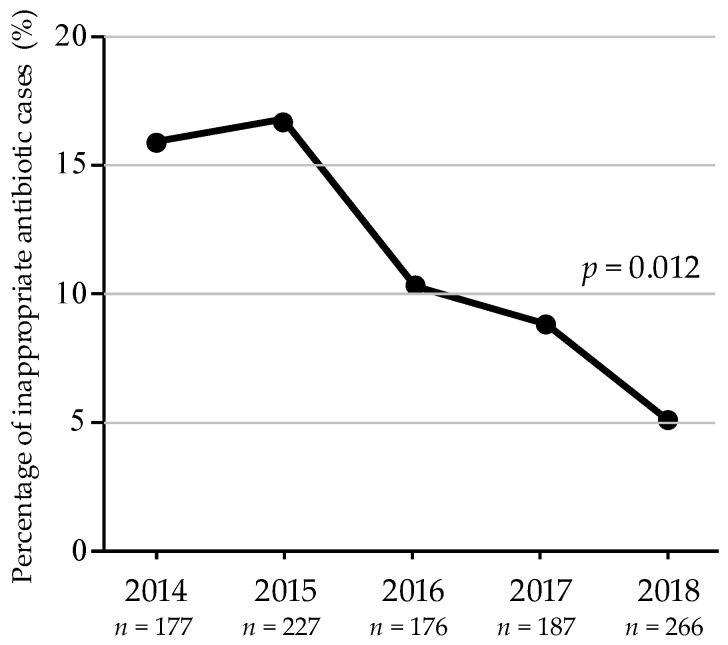
The percentage of inappropriate antibiotic cases among the patients audited by pharmacists between 2014 and 2018.

From January 2014 to December 2018, a total of 1033 urological inpatients, who were treated with broad-spectrum antimicrobials, were audited by pharmacists. Of these 1033 patients, 118 patients (106 male and 12 female), who were identified as having received inappropriate antibiotics, were discussed in the AST meetings. Figure 1 shows the percentage of inappropriate antibiotic cases identified by the pharmacists from the total count of urological patients each year. The percentage of cases identified as having used antibiotics inappropriately significantly decreased during the study period (*p* = 0.012).

Table 1 shows the details of infectious diseases in 118 patients. The most common diseases were pyelonephritis (*n* = 55, 46.6%), followed by indefinite infection (*n* = 18, 15.3%), febrile neutropenia (*n* = 14, 11.9%), and wound infection (*n* = 5, 4.2%). Of the 118 patients discussed in the AST meetings, 28 patients (23.7%) were assessed as requiring drainage by urologists. Statistical analyses showed that the number of indefinite infections significantly decreased over a five-year period (*p* = 0.033).

Table 2 shows the antibiotics discussed in the meetings; other antibiotics not listed in Table 2 were not prescribed for urological patients. Antipseudomonal penicillin (*n* = 67, 56.8%) was most frequently prescribed, followed by carbapenems (*n* = 25, 21.2%), antipseudomonal cephalosporins (*n* = 16, 13.6%), and anti-MRSA antibiotics (*n* = 10, 8.5%). The number of cases who had been prescribed antipseudomonal penicillin inappropriately significantly decreased (*p* = 0.017).

### 3.2. Reasons for Intervention

Table 3 shows the reasons for AST intervention. The representative reasons for intervention included the need for de-escalation (*n* = 35, 29.7%), dose optimization (*n* = 25, 21.2%), inappropriate selection of antibiotics (*n* = 20, 16.9%), and patients for whom no cultures had been submitted (*n* = 11, 9.3%).

### 3.3. Microbiological Culture Collections

Table 4 shows blood, urine, or drainage culture collections during the study period. Blood and drainage culture collections significantly increased during the study period (*p* = 0.009 and *p* = 0.035, respectively).

## 4. Discussion

The AS concept is spreading nationwide in Japan, partly due to the AMR National Action Plan [7]. The concept aims at controlling antimicrobial use, decreasing the number of AMR bacterial strains and, importantly, reducing the unnecessary use of broad-spectrum antibiotics [8]. In collaboration with ICDs, the AST plays a major role in managing antimicrobial use to control infectious diseases due to AMR bacterial strains. 

AST and ICD interventions have been studied before. Honda et al. reported that the initiation of adequate empiric antimicrobial therapy had important implications for AS even in the elderly population in Japan [9]. Other studies have also discussed AST and infectious disease control from the point of view of physicians, pharmacists, or clinical microbiologists [6,10,11,12].

Urologists have an important role in infectious disease management of patients with UTI, urosepsis, or drainage for obstructive disease such as a stone-related obstructive UTI. To assess the indications for drainage or stenting in the urinary tract, ASTs need to collaborate with surgeons, including urologists [13]. They can decide to carry out drainage using ureter stents, urethral catheters, or renal fistulas, as necessary, and ensure that they do not miss an opportunity to collect culture and choose the appropriate antibiotics. Proper drainage improves renal function, and improved renal function makes antibiotic treatment more effective [14]. The timing of surgical intervention should not be delayed by performing ineffective, empiric antibiotic treatments for infection, which is one of the risk factors for lower survival in sepsis [15].

Our data demonstrate that intervention by the AST significantly decreased both the percentage of inappropriate antibiotic use cases and the number of cases for intervention, specifically, the number of indefinite infections over the five-year study period (*p* = 0.012 and *p* = 0.033, respectively). Usually, this kind of study tends to be conducted in an internal medicine department where infectious disease management tends to be limited to the selection of an antimicrobial regimen and microbial laboratory examination. However, infectious diseases should be often treated by drainage, including surgical drainage performed by surgeons. Recently, studies conducted by urological departments have been published. Spooremberg et al. demonstrated that an earlier switch from intravenous to oral treatment led to more favorable patient outcomes and lower healthcare costs [16]. Doernberg, et al. conducted quasi-experimental historical controls with a six-month retrospective period versus a six-month intervention period for asymptomatic bacteriuria and UTI in patients in a long-term care facility setting, with 104 antibiotic prescriptions for UTI. They demonstrated: (1) weekly prospective audit and feedback from the AST over a six-month period resulted in decreased antibiotic use, (2) many lost opportunities for intervention were identified, and (3) no significant effect was noted on resistant organisms or detection of *Clostridioides difficile* [17]. Dik et al. studied an AST intervention cohort vs. a historic control cohort by time series analysis for antibiotic treatments in urological wards (114 intervention and 357 control cases) and showed significant reduction of antimicrobial consumption for all patients, decreased length of hospital stay, and an unchanged outcome for patients with severe underlying diseases, such as cancer [18]. The AST recommended antimicrobial treatment adjustment/de-escalation to appropriate therapy in the majority of bacteremic UTI patients, and the results showed a significantly lower mortality rate in de-escalation groups compared to those with no antimicrobial change [19]. AST intervention was an independent variable related to clinical cure, but no economic impact was seen in a retrospective study of UTI patients with extended spectrum beta-lactamase (ESBL)-producing bacteria [20]. Another retrospective cohort study of complicated UTI cases demonstrated significant reduction in the duration of antibiotic treatment and reduced length of hospitalization [21]. A representative review by a urological AST concluded that well-designed ASTs help reduce treatment duration, time-to-switching-to oral-antibiotics, and total antibiotic prescription, with an optimal assessment timing of approximately 24–48 h. AST programs are also useful for the education and feedback for physicians [22].

We recommend that ASTs need to include urologists or surgeons to help avoid biases towards considering antimicrobial selection only for intervention and focusing on only broad-spectrum agents, such as carbapenems, rather than on surgical intervention. These biases negatively affect antimicrobial use and possibly patient outcomes, especially in surgical departments.

There are several limitations of the present study. First, this is a retrospective study with a small number of cases. Second, we had no available data on drainage procedures such as urinary tract stenting or percutaneous drainage. Third, no control groups were included. These limitations will be addressed in our future studies.

## 5. Conclusions

Our study was conducted with a well-established AST over five years [6]. Statistical analyses showed that the percentage of cases of inappropriate broad-spectrum antibiotic use and interventions for indefinite infections had significantly decreased, whereas the number of blood or drainage culture submissions had significantly increased over the five-year study period. These results suggest that urologists have probably become more familiar with antibiotic treatments based on evidence such as microbiological culture results. AST intervention might help to prevent inappropriate antibiotic use in surgical departments. Further studies are necessary for follow-up.

## Figures and Tables

**Table 1:** Urological infectious diseases discussed in the antimicrobial stewardship teams (AST) meetings from 2014 to 2018.

Infectious disease	2014–2018 (*n* = 118)	2014 (*n* = 29)	2015 (*n* = 39)	2016 (*n* = 19)	2017 (*n* = 17)	2018 (*n* = 14)	*p*
Disease that require drainage	28	4	7	8	5	4	0.87
Pyelonephritis	55	9	15	10	10	11	0.63
Indefinite infection	18	6	6	3	1	2	0.033
Febrile neutropenia	14	1	9	4	0	0	0.17
Wound infection	5	2	1	1	1	0	-
s/o peritonitis	4	4	0	0	0	0	-
Acute bacterial prostatitis	3	1	0	2	0	0	-
CRBSI	2	1	1	0	0	0	-
Bacteremia	2	2	0	0	0	0	-
Acute prostatitis	2	0	2	0	0	0	-
Others	16	3	5	1	6	1	0.74
Total cases	121 ^a^	29	39	21	18	14	

s/o, suspect of; CRBSI, catheter-related blood stream infection. ^a^ As some patients had > 1 infectious disease, the total count exceeded 118.

**Table 2:** Antibiotics prescribed for patients listed in Table 1.

Antibiotics	2014–2018 (*n* = 118)	2014 (*n* = 29)	2015 (*n* = 39)	2016 (*n* = 19)	2017 (*n* = 17)	2018 (*n* = 14)	*p*
Antipseudomonal penicillins	67	18	16	13	12	8	0.017
Carbapenems	25	4	9	3	4	5	0.94
Antipseudomonal cephalosporins	16	7	7	1	0	1	0.11
Fluoroquinolones	4	0	1	1	1	1	-
Anti-MRSA agents	10	1	6	2	0	1	0.49
Total cases	122 ^a^	30	39	20	17	16	

^a^ As some patients had > 1 prescribed antibiotic, the total count exceeded 118.

**Table 3:** Reasons for AST intervention.

Reason for intervention	2014–2018 (*n* = 118)	2014 (*n* = 29)	2015 (*n* = 39)	2016 (*n* = 19)	2017 (*n* = 17)	2018 (*n* = 14)	*p*
De-escalation	35	5	12	7	7	4	0.50
Dose optimization	25	10	8	4	0	3	0.083
Inappropriate selection of antibiotics	20	5	6	4	3	2	0.083
No cultures submitted for pathogen identification	11	6	3	0	1	1	0.17
Duration of antimicrobial therapies	10	0	2	0	5	3	0.17
Escalation	4	3	1	0	0	0	-
Unknown focus	2	0	0	0	1	1	-
Others	20	6	5	5	2	2	<0.001
Total cases	127 ^a^	35	37	20	19	16	

^a^ As some patients had > 1 reason, total count exceeded 118.

**Table 4:** Microbiological culture collections (blood culture sets/1000 patient-days, urine or drainage cultures/1000 patient-days).

Sample Type	2014	2015	2016	2017	2018	*p*
Blood culture	15.8	19.9	22.3	22.3	26.6	0.009
Urine culture	14.4	25.9	35.4	36.1	27.4	0.24
Drainage culture	0.11	0.46	0.39	0.51	0.89	0.035
